# Review of Workplace Based Aerosol Sampler Comparison Studies, 2004–2020

**DOI:** 10.3390/ijerph18136819

**Published:** 2021-06-25

**Authors:** James Hanlon, Karen S. Galea, Steven Verpaele

**Affiliations:** 1 IOM, Research Avenue North, Riccarton, Edinburgh EH14 4AP, UK; karen.galea@iom-world.org; 2 Nickel Institute, Rue Belliard 12, 3rd Floor, B-1040 Brussels, Belgium; sverpaele@nickelinstitute.org

**Keywords:** inhalable, respirable, thoracic, aerosol, particulates, comparison, performance, inter-sampler, EN13205, workplace

## Abstract

We provide a narrative review on published peer-reviewed scientific literature reporting comparisons of personal samplers in workplace settings published between 2004 and 2020. Search terms were developed for Web of Science and PubMed bibliographic databases. The retrieved studies were then screened for relevance, with those studies meeting the inclusion criteria being taken forward to data extraction (22 studies). The inhalable fraction was the most common fraction assessed with the IOM sampler being the most studied sampler. The most common workplace environment where samplers had been compared was that where metals/metalloids were present. The requirements of EN13205 standard (Workplace exposure. Assessment of sampler performance for measurement of airborne particle concentrations) have also been considered, with these requirements not currently being met, or at least referred to, in the included published literature. A number of conclusions have been drawn from this narrative review. For studies that reported correction factors, no discernible trends could be identified. Correction factors also varied between samplers and settings, with correction factors varying from 0.67 for Button/IOM in agriculture settings to a correction factor of 4.2 for the closed face cassette/IOM samplers in aluminium smelters. The need for more detailed and informative data sharing from authors is highlighted, providing more context to both the sampling strategy and methodology, as well as the data analysis. It is recommended that the requirements of EN13205 are taken into account when designing sampler comparison studies at the workplace and that these are also reported. It is also considered that there is a need for a clear standardized workplace sampler comparison protocol to be developed, which can be used by the research and occupational hygiene community to allow more robust and transparent assessment of aerosol samplers and better-quality evidence for use by industrial hygienists, epidemiologists, and occupational safety specialists alike.

## 1. Introduction

Exposure to hazardous substances may occur in the workplace in the form of aerosols. The term ‘aerosol’ is used to describe any suspension of particles in air, and most aerosols consist of a wide range of particle diameters. The British Medical Research Council definition of the respirable aerosol fraction (those particles with a median aerodynamic diameter of 5 μm collected with a 50 % efficiency) was the first recognized internationally [1]. In 1989, new criterions for aerosol fractions were proposed by Soderholm [2] and international collaboration led to the agreement on the definitions of health-related aerosol fractions in the workplace, defined as inhalable, thoracic, and respirable, that relate to the region of the respiratory tract where they are most likely to deposit. The convention for these size fractions is described in ISO 7708 [3].

In ISO 7708, the inhalable convention target sampling curve for instruments that collect the inhalable fraction for wind speeds of below 4 m/s averaged over all of the wind directions is described. For this convention, the equation to be used the airborne particle percentage (E_i_) which have the aerodynamic diameter (D; µm) for collection is: E_i_ = 50 (1 + exp[−0.06 D]). For the thoracic convention, the target sampling curve is the percentage of the inhalable convention to be collected at an aerodynamic diameter using a cumulative log-normal distribution. The median for this distribution is 11.64 µm with a 1.5 geometric standard deviation (GSD). The respirable convention target sampling curve is the percentage of the inhalable convention for collection at an aerodynamic diameter using a cumulative log-normal distribution. The median of this distribution is 2.5 µm with a 1.5 GSD.

Regulatory bodies and research institutions have increasingly lowered occupational exposure limit values (OELVs) in response to increased understanding of health effects and routes of exposure [4,5,6]. However, these OELVs are chosen with the health of the worker in mind rather than technical or analytical feasibility [7]. An additional complicating factor is that research has recognized that differently sized fractions of particles have different health impacts, which has required refining of the sampling process to detect air concentration levels of each fraction. Low OELVs and the need to differentiate between size-selective fractions present a unique challenge to industrial hygienists, epidemiologists, and occupational safety specialists. All aspects of determining the presence of a hazardous substance, including sampling, sample dissolution, and the analytical methods themselves, must be optimized to attain these lower limits precisely and accurately [8].

A recent survey of aerosol sampling heads used within the metals industry (personal communication, S Verpaele) was done in parallel to a survey of European laboratories concerning the methods used for the determination of nickel in workplace air [9]. This survey revealed a wide variety of inhalable, thoracic, and respirable samplers as being commonly used (Appendix A).

In April 2019, the Nickel Institute, a global association of primary nickel producers, held a meeting with interested parties regarding the development or adaptation of existing sampling trains to measure low levels of metals and metalloids in the workplace. The parties involved agreed on the need for an international sampler comparison study. The main objective of this international study is to compare currently used (and validate any newly developed) personal samplers for measuring particulate related exposure (and more specifically metals and metalloids) in workplace settings. Sampler efficiencies for relevant aerosol size fractions of those samplers currently on the market will also be included in this study [10].

Within the framework, two stakeholder groups were created. The first is the Sampler Comparison Industry Group (SCIG) including industry stakeholders. The tasks of this group are related to foreseeing budget, making sure the industry is represented and their needs identified and that they engage with their members and individual companies about the project. The second is the Sampler Comparison Scientific Group (SCSG) including those involved in research institutes and universities. In this group, the project plans are established and executed. An important task for both groups is to identify worldwide research grants that can be applicable to this project. The first project granted within this framework was a WorkSafe British Columbia (Canada) project to compare the most commonly used sampling techniques with more advanced sampling techniques for metals and metalloids in North America. In parallel, protocols for testing respirable and inhalable samplers in laboratory test chambers are being developed and this literature review, which aims to summarise the literature describing sampler comparison studies in workplace settings along with a further review focused on laboratory comparison and efficiency studies are being executed. The long-term aim of the overarching international sampler comparison study is to ensure exposure data which is used on epidemiological studies is both precise and aligned for use in the setting of OELVs.

Various historic studies have been published comparing the performance of different samplers [11,12,13,14,15,16,17,18,19,20,21,22,23]. Findings suggest that using different samplers can result in significant differences in the observed particle concentration. Wind velocity and direction, inlet size, geometry, orientation, aerosol particle size, electrical charge, particle bounce properties, the sampler conductive properties along with other factors have been identified to affect the performance of samplers [24]. The varying performance of different sampling devices may cause a degree of uncertainty when using the sampling results to check compliance with regulatory limits, or when the data are used for risk assessment and management purposes.

The purpose of the EN13205 standard (which was first published in 2002) is to allow both manufacturers and users to use a consistent approach for sampler validation and to provide a framework for assessing sampler performance in adherence to standards EN481 [25] and EN482 [26]. Since its first publication in 2002 (EN 13205:2002), this standard has been updated on one occasion in 2014 (EN 13205:2014). The current standard consists of six separate parts: Part 1 which sets the general requirement, Part 2 for performing laboratory based tests which is based on sampling efficiency determination, Part 3 which sets out the requirements for the analysis of sampling efficiency data, Part 4 which sets out the requirements for performing laboratory performance tests for concentration comparisons, Part 5 which sets out the requirements for workplace-based sampler comparison and performance tests, and Part 6 which concerns the transport and handling tests.

The requirements, relevant to this review are those in Part 5 of the standard. Some general requirements are set out for personal samplers for inhalable, thoracic, and respirable aerosol fractions and static sampling with respect to the location of the samplers during the test. For the performance of samplers in the workplace, a comparison between concentrations sampled from a specific workplace should be performed between a candidate sampler and a previously validated sampler.

EN 13205:2014 describes a number of requirements for performing workplace-based sampler comparisons and these are summarised in Table 1.

This manuscript reports on a narrative review performed of the peer-reviewed scientific literature on personal samplers used in workplace settings published between 2004 and 2020, with a focus on those that are used for sampling metals and metalloids. The literature has also been compared to the requirements of EN13205, to identify potential gaps in the experimental requirements with respect to this standard. A narrative review of laboratory-based sampler comparison studies will be published separately.

## 2. Methods

### 2.1. Search Strategies

The search strategies discussed in this section are for both workplace and laboratory-based sampler comparison study reviews.

The literature search was conducted in Web of Science and PubMed for studies published between 2004 and 2020 using the following search terms: (compare OR comparison* OR evaluat* OR efficiency OR performance) AND sampler* AND (aerosol OR particulate).

The inclusion criteria for the screening process included: articles published in English, field (workplace) comparison studies, laboratory/wind tunnel-based comparison studies, contained a comparison of at least two particulate samplers, and sampler efficiency and performance tests. The exclusion criteria used for the screening process were articles that included samplers that cannot be used in the assessment of personal workplace exposure, vapour/gas samplers, bio aerosol samplers, direct reading instruments, and studies and devices that were reported to be used for assessing environmental air quality (particulate matter samplers). Articles that did not include a sampler comparison were also excluded. The inclusion/exclusion criteria were performed firstly on the title and abstracts of the article, followed by screening of the full text of articles.

**Figure 1:**
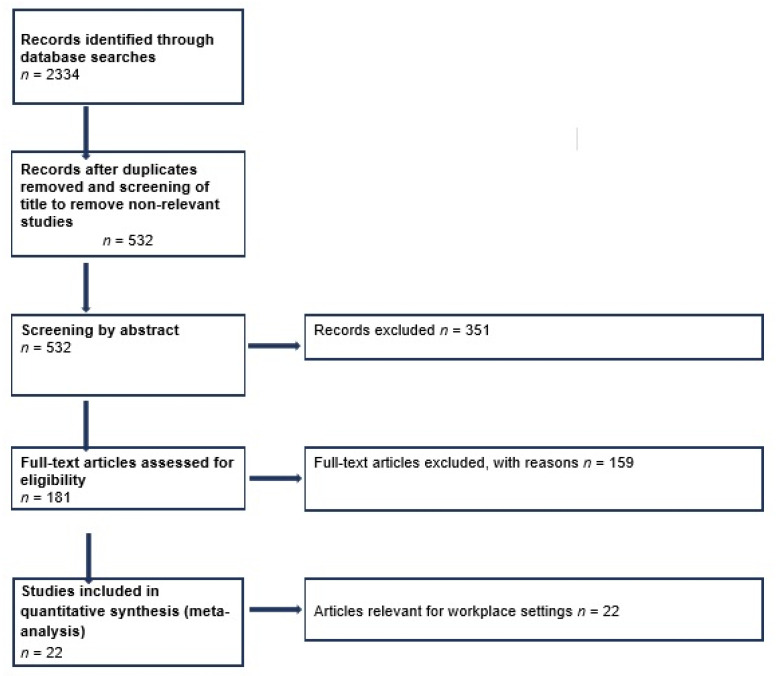
PRISMA Flow Diagram for sampler comparison review.

Figure 1 summarizes the review process. The initial search retrieved 2334 publications. These were subsequently reduced to 532 publications after duplicates were removed and title screening. The number of relevant publications were then reduced to 181 publications following abstract screening. These were then subsequently screened for relevance by full text screening. Twenty-two publications were identified as relevant as sampler comparison studies in workplace settings and were subject to data extraction.

### 2.2. Data Extraction and Collection Process

Data from the relevant publications were extracted by using separate bespoke templates, which were set up using DistillerSR^®^ (Evidence Partners, Ottawa, ON, Canada).

For the purpose of the workplace-based review, the template collected information on (where available): the samplers assessed, assessed aerosol fractions, article setting, location of the sampler, filter materials, laboratory analysis, activities/processes, dust assessed, sampling periods and measurements, sampling flow rates, summary of the results and conclusions, correction factors/ratios, and limitations of the publication (where stated by the authors).

Studies have been reviewed against the required criteria in the EN 13205:5 [27] standard (Table 1). No further consideration has been given for the article design, article robustness, or the article quality.

## 3. Results

### 3.1. Samplers and Size Fractions Studied

A variety of samplers have been investigated in the identified sampler comparison publications in workplace settings (22 studies), which are summarized in Table 2. Over half of the articles (68%) included a comparison of the IOM inhalable sampler. The 37-mm closed-face cassette (CFC) for total fraction was the next most studied sampler (55%). The Button (inhalable) sampler was studied on five occasions (5 studies; 23%) followed by the Respicon sampler (4 studies; 18%), which is capable of measuring multiple aerosol fractions. In some studies, the samplers have been referred to as different sample names, for example the IOM head with the PU foam [28] is also referred to as the IOM dual sampler [29]. In this instance this is referred to as the IOM dual sampler hereafter. In other cases, the samplers are referred to as noted in the article.

For the fractions measured, samplers assessing the inhalable fraction was most commonly reported (68% of studies), with the IOM head being the most commonly reported, as previously mentioned [30,31]. The IOM head has been compared with other inhalable samplers, e.g., the Button sampler [32] and with the CIP 10-l sampler [33]. Inhalable samplers less commonly compared included the Italian Cone Sampler [34] which was compared with the IOM sampler and the Gesamtstaub-Probenahmesystem (GSP) sampler [35,36] which was compared with the Button, IOM sampler, 37-mm CFC, and the CIP-10l samplers [35] as well as the 37-mm CFC sampler [36].

Under half of the retrieved studies assessed the respirable fraction (32%). This includes the Higgins-Dewell cyclone [37,38], the Aluminium SKC cyclone [39,40], and the GK2.69 cyclone [29]. The thoracic fraction has only been assessed in two of the retrieved studies (>9%), with the GK2.69 cyclone being used in both [38,40]. The ‘total’ aerosol fraction, based on if the study stated the ‘total’ fraction was assessed has been assessed in over half of the retrieved studies (52%), with the 37-mm CFC [33] being used in such instances.

In 27% of the publications, multifraction samplers have also been used and compared with differing samplers. The IOM dual sampler, which measures both the inhalable and respirable fractions has been used in three publications [28,29,37]. In addition, two publications [40,41] have used the Respicon sampler to analyse the inhalable, respirable and thoracic fractions and one publication has used the Respicon sampler to analyse the inhalable and thoracic fractions [42].

**Table 2:** Samplers assessed in workplace settings (note—names listed as stated in publication except for the IOM dual sampler).

Aerosol Fraction	Sampler	Articles (*n* = 22)	References
Inhalable	IOM Sampler	15	[28,29,30,31,32,33,34,35,38,39,40,42,43,44,45]
	Disposable inhalable aerosol sampler (DIAS)	1	[45]
	Button sampler	5	[28,32,33,35,43]
	Millipore (25 mm and 37 mm) open-faced cassette	1	[46]
	37-mm closed-face cassette (CFC) with ACCU-CAP	2	[33,35]
	GSP sampler	2	[35,36]
	Italian Cone Sampler (Zambelli s.r.l)	1	[34]
	Seven hole sampler	1	[46]
	Polish sampler	1	[46]
	CIP 10-l	1	[33,35]
	Prototype sampler (*no name supplied*)	2	[30]
Respirable	GK2.69 cyclone	1	[37]
	Higgins-Dewell cyclone	2	[38]
	Aluminium SKC cyclone	3	[39,40,43]
	IOSH cyclone	1	[41]
Thoracic	GK2.69 cyclone	2	[38,40]
Total	Closed face 37-mm cassette (37-mm CFC)	12	[31,32,33,36,38,39,42,43,44,47,48,49]
	Dual IOM sampler	4	[23,28,29,37]
Multi-fraction	Respicon	4	[40,41,42,46]
	Novel three stage sampler	1	[41]
	8-stage Sierra cascade impactor	1	[39]
	10-stage MOUDI impactor (compared to personal samplers)	1	[39]
	Two stage cascade impactor model PM 10/4, Dekati Ltd.	1	[28]

### 3.2. Workplace Sampler Comparison Settings

The table in Appendix B, summarises the samplers used, the cassette materials used, sampling substrate materials, sampling type, sampler flow rates, and also if particle size distribution has been considered. The table in Appendix C further summarises the sampling and analysis performed.

The most common workplace setting involved sampling metals/metalloids. Nine studies were identified in metals/metalloids workplace settings (Appendix C) including for aluminium [39], lead [41] and alloys such as copper-beryllium [36]. Five studies were identified as comparing samplers in wood processing environments [32,33,34,35,40]. Agriculture settings accounted for two studies [30,43]. Road paving also accounted for two studies [44,47]. Brick manufacturing [37], cement plants [38], and shipbuilding yards [48] were subject to one article comparison.

#### 3.2.1. Metal/Metalloids Settings

In an aluminium smelter sampling for aluminium dust with a Respicon sampler (flow rates of 2.66, 0.33 and 0.11 L min^−1^ through the orifices), 25-mm CFC and an IOM inhalable sampler compared at sampler flow rates of 3.1 L min^−1^ and 2.0 L min^−1,^ respectively, the Respicon sampler under sampled both the aerosol mass and the water-soluble fluoride (fluoride in the collected air mass) compared to the IOM sampler for the inhalable fraction [42]. The Respicon also under sampled when the concentration was higher than 1.35 mg/m^3^ but over sampled at lower mass concentrations. The Respicon for the thoracic fraction and CFC samplers collected similar aerosol masses, however for the water-soluble fraction, the ratio was 1.19.

Two articles were identified for sampling beryllium dust. These sampler comparisons were performed in a magnesium foundry and aluminium smelters [39] and also in the processing of copper–beryllium [36]. The CFC 37-mm sampler has been used in both these studies for measuring the ‘total’ dust. In Dufresne et al. [39] where both personal and static (area) samples were collected, it was found that the median of the median (as reported by the authors; no explanation in the article) beryllium concentration ratios from sampling heads decreased in the following order when the sampling heads were at a fixed station: IOM sampler (inhalable) (1.00) > Sierra cascade impactor which was assessing its performance for airborne beryllium (0.69) > 37-mm CFC (‘total’ dust) (0.64) > MOUDI impactor for assessing its performance for airborne beryllium (0.54) > SKC Aluminium cyclone (respirable) (0.19). Dust median concentrations varied between samplers and their locations. The IOM sampled between 0.78–6.10 mg/m^3^ at fixed stations and between 2.24–34.0 mg/m^3^ for breathing zone samples. The 37-mm CFC sampled between 0.24–4.20 mg/m^3^ at fixed stations and between 0.73–7.05 mg/m^3^ for breathing zone samples. The SKC cyclone sampled between 0.05–1.03 mg/m^3^ at fixed stations and between 0.25–1.65 mg/m^3^ for breathing zone samples. In the copper-beryllium processing setting [36], a linear relationship is obtained for beryllium concentrations sampled using the CFC sampler (‘total’ fraction) and the GSP sampler (inhalable fraction), with conversion factors as a result of this article being reported in Table 3. The geometric mean (GM) masses collected as reported by the authors were 5.88 ng/m^3^ and 18.55 ng/m^3,^ respectively, for the CFC and GSP sampler for personal samplers and 7.41 ng/m^3^ and 17.63 ng/m^3,^ respectively, for static samples.

Only one article was identified for copper dust sampling [45]. This article compared a disposable version of the original IOM head (sampler is referred to as a disposable inhalable aerosol sampler (DIAS)) that had been developed with an IOM sampler for the inhalable fraction in a copper electro refinery at a flow rate of 2.0 L min^−1^. The DIAS sampler was developed by L’Orange et al. [50] and has also been assessed in agricultural environments (Anthony et al. [30]). Both personal measurements and area sampling were undertaken. The DIAS sampled higher concentrations than the IOM sampler for both personal and area sampling situations with 81% of the combined data showing the DIAS/IOM ratio above one. There were no statistically significant differences between the samplers for personal measurements (*p* = 0.478); however, this was not the case for area samples (*p* = 0.031) and the combined sampling data (*p* = 0.031). The geometric means for both the combined data set (personal and area sampling) were 26.5 µg m^−1^ for the IOM sampler and 36.1 µg m^−1^ for the DIAS sampler.

Three articles were identified for comparing samplers when sampling lead dust. Chisholm et al. [31] sampled in a bronze foundry and found that the mass-weighted sample size distributions (as discussed by the authors for filter and wall deposits) for both the IOM and 37-mm CFC filter sample size distribution did not differ significantly (*p* < 0.05) at sampler flow rates of 2.0 L min^−1^ for the inhalable fraction. Only one statistical difference in the mass-weighted size distribution for the 15 pairs for sampling lead dusts in a copper smelter was obtained. This was also the case in a lead ore concentrate mill (one IOM filter sampler statistically differed significantly) and in a lead acid battery factory where one sample also differed.

The 37-mm CFC has also been subject to an article by Lee et al. [49] to compare using filter-only CFC and a 37-mm CFC with a customized insert of a mixed-cellulose filter and an acid-digestible cellulose–acetate cassette capsule for sampling lead dust for solder manufacturing for the ‘total’ fraction. The geometric mean concentrations for the filter only 37-mm CFC were 53% lower than that of the CFC with a customized insert; however, these were comparable with the results of the filter wipe from the filter-only CFC. Tsai et al. [41] concluded that for measuring the respirable fraction for lead in a number of locations (lead powder factory, lead acid battery plant and a casting factory), the IOSH cyclone and a developed novel three-stage sampler collected similar respirable concentrations (<10% difference). However, these samplers overestimated the respirable dust concentrations by 20% for the three-stage sampler and 31% for the IOSH cyclone when compared to the Respicon sampler.

The IOM dual sampler and the GK2.69 respirable cyclone have been compared for measuring exposure to indium and dust in an indium tin oxide manufacturing facility for the respirable fraction and the IOM dual sampler has been compared with the IOM sampler for the inhalable fraction [29]. The sampler flow rate for the IOM sampler was 2.0 L min^−1^ and 4.2 L min^−1^ for the cyclone. The dual IOM sampler had good agreement with the IOM sampler for the indium inhalable fraction (concordance correlation coefficient of 0.997), but a lower agreement was obtained for inhalable dust (concordance correlation coefficient of 0.866 with a mean bias of −146.9 mg/m^3^). The geometric mass concentration for inhalable indium collected for the dual IOM sampler was 36.5 µg/m^3^ and 35.1 µg/m^3^ for the IOM sampler. The dual IOM sampler had a geometric mass concentration of 329.1 µg/m^3^ compared to 503.9 µg/m^3^ for the IOM sampler. For the respirable indium fraction, there was a better agreement for respirable indium compared to respirable dust (concordance correlation coefficients of 0.932 and 0.777, respectively) for the dual IOM sampler. The geometric mean of mass concentration obtained for the respirable indium fraction was 10.9 µg/m^3^ for the dual IOM sampler and 8.4 µg/m^3^ for the respirable cyclone and 118 µg/m^3^ for and 76.6 µg/m^3^, respectively, for respirable dust.

Only one article was identified for comparing samplers when sampling tin dust. This article compared using filter-only CFC and a 37-mm CFC with a customized insert of a mixed-cellulose filter and an acid-digestible cellulose-acetate cassette capsule for sampling ‘total’ tin dust for solder manufacturing at a sampler flow rate of 2.0 L min^−1^ [49]. The geometric mean concentrations for the filter only 37-mm CFC were 32% lower than that of the CFC with a customized insert; however, these were comparable when the results of the filter wipe from the filter-only CFC was considered.

One article [28] compared the IOM sampler, the IOM dual sampler, and the Button sampler (metal smelter and foundry) for the inhalable fraction. In the metal plants, the IOM sampler collected higher concentrations of inhalable dust concentrations than the Button sampler. The IOM dual sampler collected the most dust for the inhalable fraction.

#### 3.2.2. Agriculture

In agriculture settings, Anthony et al. [30] compared the DIAS at a sampler flow rate of 10 L min^−1^ and the IOM sampler at a sampler flow rate of 2 L min^−1^ for the inhalable fraction in a swine farrowing room using static measurements. The mean mass concentration collected by the DIAS sampler was 0.03 mg/m^3^ higher than the IOM sampler when comparing paired data (*n* = 36). This difference was insignificant (*p* = 0.16) with a high correlation also obtained between the mass concentrations of the two samplers (Spearman correlation coefficient of 0.85). The mean mass concentration of inhalable concentrations collected by the DIAS sampler was also 0.03 mg/m^3^ higher than that collected by the IOM sampler.

Reynolds et al. [43] used field trials (along with laboratory trials) for the IOM sampler (sampler flow rate of 2.0 L/min), the Button sampler (sampler flow rate of 4.0 L/min). and the 37-mm CFC (sampler flow rate of 2.0 L/min) for the inhalable fraction and the SKC Aluminium cyclone for the respirable fraction (sampler flow rate of 2.5 L/min). In swine, chicken, turkey, and dairy environments, the IOM sampler collected more gravimetric mean dust (2.97 mg/m^−3^ for swine, 2.66 mg/m^−3^ for chicken, 3.52 mg/m^−3^ for turkey, and 0.32 mg/m^−3^ for dairy) followed by the CFC (1.52 mg/m^−3^ for swine, 1.59 mg/m^−3^ for chicken, 2.05 mg/m^−3^ for turkey, and 0.12 mg/m^−3^ for dairy) and then the Button sampler (1.87 mg/m^−3^ for swine, 1.97 mg/m^−3^ for chicken, 1.83 mg/m^−3^ for turkey, and 0.17 mg/m^−3^ for dairy). The SKC cyclone collected the least gravimetric mean dust (0.20 mg/m^−3^ for swine, 0.39 mg/m^−3^ for chicken, 0.43 mg/m^−3^ for turkey, and 0.08 mg/m^−3^ for dairy). This is not unexpected as it is assessing the respirable fraction which is a subcomponent of the inhalable fraction.

#### 3.2.3. Brick Manufacturing

One article has been identified for comparing samplers for dust in brick manufacturing. The respirable and inhalable fractions were assessed using a JS Holdings Higgins-Dewell plastic cyclone (respirable) and an IOM dual–fraction sampler (respirable and inhalable fractions) with a plastic cassette at sampler flow rates of 2.0 L min^−1^ and 2.2 L min^−1,^ respectively [37]. Static measurements consisting of 72 pairs were collected. The Higgins-Dewell cyclone (0.07–26.85 mg/m^3^) measured around 2.5 times than the respirable fraction of the IOM dual sampler (0.07–7.69 mg/m^3^). After log transformation, the Higgins-Dewell cyclone measured concentrations on average 1.9 times higher than that of the IOM dual sampler for the respirable fraction.

Measurements were divided into three groups for the most dominate exposure at the location where the measurements were taken. These three groups were clay particulates, mixed exposure and silica dust. Correlation for the Higgins-Dewell cyclone and IOM dual-fraction sampler were best obtained measuring silica dust (Pearson correlation coefficient (r_p_) of 0.88 (0.63–0.96)) followed by clay particulates in the ‘mixed’ group (r_p_ of 0.82 (0.59–0.93)) and then r_p_ of 0.74 (0.65–0.85) for the ‘clay particulates’ group.

It was concluded by this study that the IOM dual fraction sampler when compared with the cyclone for sampling the respirable fraction in the brick industry may lead to significant measurement errors. The dual fraction sampler also under sampled clay particulates by up to 50%.

#### 3.2.4. Cement Plants

Notø et al. [38] investigated samplers using personal measurements in production, cleaning, maintenance and in laboratory/administration activities in cement plants in eight countries, with the aim of establishing relationships between different aerosol fractions. The samplers compared were the Higgins-Dewell cyclone (respirable fraction), GK 2.69 cyclone (thoracic fraction), 37-mm CFC (total fraction), and the IOM sampler (inhalable fraction) at sampler flow rates of 2.2 L min^−1^, 1.6 L min^−1^, 2.0 L min^−1^, and 2.0 L min^−1^, respectively. It is worth noting that the authors are not comparing ‘like for like’ samplers and the samplers are assessing different fractions.

In this workplace setting, the ratios between the samplers were not constant for the measured concentration ranges. The median ratios for the respirable fraction, ‘total’ dust and the inhalable fraction relative to the thoracic fractions were 0.51, 2.4, and 5.9. Most of the obtained regression coefficients were different than one. The respirable/thoracic (median ratio of 0.51), respirable/total (0.20), respirable/inhalable (0.09), thoracic/total (0.41), thoracic/inhalable (0.17), inhalable/thoracic (5.9), and the inhalable/total (2.2) ratios decreased as the aerosol concentration increased. The thoracic/respirable (mean ratio of 2.0), total/respirable (4.9), total/thoracic (2.4), total/inhalable (0.45), and the inhalable/respirable (mean ratio of 11.8) ratios increased as the aerosol concentration increased.

#### 3.2.5. Road Paving

Road paving settings involving three activities were sampled using a 37-mm CFC (‘total’ fraction) both with a cellulose-acetate cassette insert and without the insert and with an IOM sampler for the inhalable fraction [44]. The activities included the removal of asphalt concrete pavement, asphalt overlay, and asphalt paving with 54 worker samplers and 108 mannequin samples obtained. A 1:1 relationship existed between all the samplers when exposure was only to asphalt fumes. However, when dust or old asphalt millings (confounders) were present in the workplace settings, significant differences between the two samplers were measured. The ratios for the IOM sampler to total particulate was 1.02 for asphalt over asphalt pavements, 1.37 for milling asphalt and 1.41 for asphalt paving. Deygout et al. [47] compared the 37-mm CFC (‘total’) and the IOM inhalable sampler for sampling activities in the road paving industry which included dense and open asphalt concrete and chipping operations. In the collection of organic aerosols emitted from hot bitumen fumes, there was no statistical difference between the 37-mm CFC and the IOM sampler (*t*-test, *p* = 0.92).

#### 3.2.6. Rubber Manufacturing

In a rubber manufacturing setting, De Vocht et al. [46] compared the use of a number of samplers for measuring the inhalable fraction for rubber dust exposure in the Netherlands, Poland, and Germany for mixing, milling, and curing activities. The CALTOOL (20 L min^−1^ flow rate), seven hole sampler, PAS-6 sampler, 25-mm and 37-mm Millipore filter cassettes, IOM sampler, and the ‘Polish’ sampler have been compared (all samples used a flow rate of 2 L min^−1^). The Respicon sampler was also used for Particle Size Distribution (PSD) measurements. In this setting, all the samplers apart from the IOM sampler under sampled the inhalable fraction compared to the CALTOOL device. Relative to the CALTOOL device, the geometric means were 1.04 for the IOM sampler, 0.68 for the seven-hole sampler, 0.65 for the 25-mm Millipore filter cassette, 0.67 for the Millipore 37 mm cassette, 0.73 for the PAS-6, and 0.54 for the ‘Polish’ sampler. Further investigation on the Polish sampler has been performed during the review; however, no further information was available.

Significant differences were observed between the performances of inhalable samplers in different departments in the European rubber industry. The authors conclude that levels of exposure cannot be compared directly for rubber dust and fumes; however, performance ratios could be used to adjust measurements in the studied samplers, which could then be mutually compared.

#### 3.2.7. Shipbuilding

Both 37-mm CFCs and 8-stage cascade impactor samplers were used for comparisons in personal measurements to ‘total’ fraction for various welding activities in a shipbuilding yard at a sampler flow rate of 2.0 L min^−1^ for both samplers [48]. For sampling manganese dust (*n* = 86), there was significant correlation between the cassette (measuring total mass) and the impactor (using stages to determine the inhalable mass) (r = 0.964, *p* < 0.001). However, the total concentration sampled by the cassette (GM of 108.1 µg/m^3^) was lower than that of the inhalable concentration sampled by the cascade impactor sampler (GM of 196.5 µg/m^3^).

#### 3.2.8. Talc Production and Peat Production

One article by Linnainmaa et al. [28] compared a number of samplers in talc and peat production for the inhalable fraction.

For mineral dust the Dekati two stage cascade impactor result was statistically significant to the IOM sampler, the IOM dual sampler and the Button sampler for sampling mineral dust. The Dekati two stage cascade impactor was also statistically different (significantly higher) than the IOM dual sampler for the respirable fraction of mineral and peat dusts. For the inhalable fraction, the IOM sampler collected the highest concentration of mineral dust. For sampling peat, the samplers provided similar results to each other.

#### 3.2.9. Wood Manufacturing

**Figure 2:**
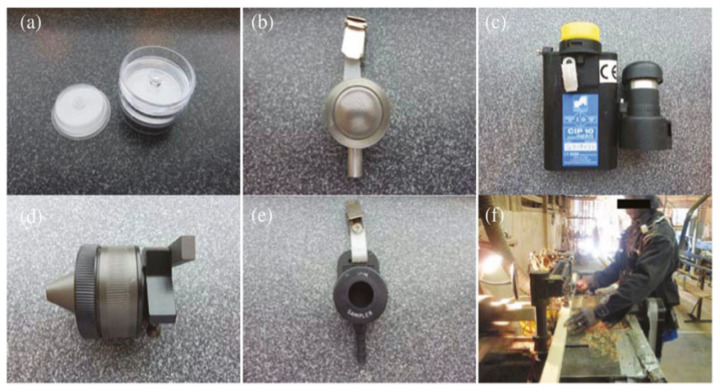
(**a**) ACCU-CAP™ sampler, (**b**) Button sampler, (**c**) CIP10-I sampler, (**d**) GSP sampler, (**e**) IOM sampler, and (**f**) wood dust sampling with the worker wearing the ACCU-CAP™ sampler and the CIP10-I sampler. From Figure 1 (Tested aerosol samplers) in Lee et al. [35] licensed under Creative Commons CC BY.

Five articles were identified comparing samplers in the wood manufacturing sector. In one article the 37-mm CFC was compared with ACCU-CAP (sampler flow rate of 2 L min^−1^), Button (sampler flow rate of 4 L min^−1^), CIP10-I (sampler flow rate of 10 L min^−1^), GSP, and the IOM (sampler flow rates of 2 L min^−1^) samplers (Figure 2) for a number of activities including cutting, debarking, and sanding, amongst others for the inhalable fraction (Lee et al., [35]). The authors concluded that for sampling wood dust, all the samplers produced similar results to each other (geometric median mass concentrations were 0.90, 0.87, 1.08, 0.95, and 1.06, respectively, for the ACCU-CAP, Button, CIP10-I, GSP, and IOM samplers. These samplers (excluding the GSP sampler) have also been compared in another article that sampled wood dust from door/window manufacturing, sawmill, production of furnishings, and staircase production for the inhalable fraction [33]. In this article, differences in the samplers were reported as in the earlier mentioned article. The IOM, CIP 10-I v1, and the ACCU-CAP samplers measured concentrations were not statistically significant (1.12, 0.94, and 0.80, respectively versus 1.0). However, all samplers sampled more dust than the 37-mm CFC, with the IOM sampler sampling twice as much as the 37-mm CFC. For sanding, cutting, and planning activities, Campopiano et al. [34] concluded that the IOM sampler at a sampler flow rate of 2 L min^−1^ and Italian Cone sampler at a sampler flow rate of 3.5 L min^−1^ for the inhalable fraction can be interchanged when small-size particles are produced. In sanding processes (where small size particles are produced), no statistically significant differences were observed for the Italian cone/IOM sampler (*p* > 0.05). Harper et al. [32] compared the 37-mm CFC, IOM sampler and the Button sampler for the inhalable fraction and found that the IOM sampler collected more than 2.16 times more dust than that of the 37-mm CFC sampler. The geometric sampler means were 48.8 mg/m^3^ for the IOM sampler, 22.2 mg/m^3^ for the CFC, and 14.0 mg/m^3^ for the Button sampler. Under sampling was observed for the Button sampler compared to the CFC sampler for aerodynamic equivalent diameters below 50 µm.

Rando et al. [40] compared the Respicon (multifraction) with the IOM sampler (inhalable fraction), the GK2.69 cyclone (thoracic dust) and the SKC Aluminium cyclone (respirable dust) for sampling industrial wood processing dust (Figure 2). The geometric mean dust levels for all plants were 1.35 mg/m^3^ for inhalable (IOM sampler), 0.31 mg/m^3^ for thoracic (GK2.69 cyclone), and 0.10 mg/m^3^ for the respirable fraction. The fraction collected by the Respicon after applying a correction factor of x 1.5 to the extrathoracic fraction was similar to that of the IOM sampler; without the correction factor the Respicon under sampled by 23% compared to the IOM sampler. For the measurement of the respirable fraction, no significant differences were observed between the Respicon and the SKC Aluminium cyclone. For the thoracic fraction, the Respicon oversampled by 48%.

### 3.3. Correction Factors

From the articles included in the full review, 68% discussed correction factors for the compared samplers (Table 3).

In agricultural settings, the conversion factors can be dependent on the sampling site with variations observed for the 37-mm CFC, Button and IOM for different poultry environments. This was also dependent on the wind speed [30]. In brick manufacturing settings, differences in particulate type was also observed between clay particulates, mixed, and sand particles for the Higgins-Dewell cyclone and the IOM dual fraction sampler [37]. For wood settings, four articles were identified with correction/conversion factors. For these articles, it is not possible to correlate a pattern in the conversion factors between the different articles due to the sampling nature. In road paving activities, the conversion factors varies depending on the nature of the activity [44]. The inhalable/total conversion factor for a CFC with an IOM sampler varies from 1.02 for asphalt over asphalt pavements to 1.41 for asphalt paving over a granular base [44].

Metal/metalloids settings were identified as the dominant setting in workplace settings. For conversion/correction factors, only four articles were identified that listed these values between samplers. These were for sampling aluminium dust, beryllium dust, copper dust, and lead dust. No discernible trends could be identified for correction factors, although the IOM sampler is most commonly used for correction factors. The IOM sampler is often considered as for inhalable sampling it is considered to be the “gold standard” [51].

**Table 3:** Reported results and correction factors for sampler comparison publications.

Setting	Aerosol Fraction Assessed/Compared	Samplers	Dust Assessed	Summary of Results	Correction Factors	Reference
**Metals**						
Aluminium smelters	Total, inhalable and respirable	IOM inhalable sampler (inhalable)	Beryllium	The median of median dust concentration ratios computed from the sampling heads at the fixed station decreased as follows: IOM (1.00) > Sierra (0.76) > 37-mm cassette (0.61) > MOUDI (0.48) > respirable (0.12). The median of median ratios of dust were: IOM (1.00) > Sierra (0.56) > 37-mm cassette (0.35) > respirable (0.06)) and Be (IOM (1.00) > Sierra (0.66) > 37-mm cassette (0.48) > respirable (0.11).	N/A	[39]
		SKC Aluminium cyclone (respirable)				
		37-mm CFC (‘total’)				
		8-stage Sierra cascade impactor				
		10-stage MOUDI impactors				
	Total and inhalable	Respicon (‘total’)	Aluminium	The Respicon under sampled the aerosol mass for the inhalable fraction compared to the IOM sampler at concentrations higher than 1.35 mg/m^3^ and oversamples at lower concentrations. The overall ratio between aerosol mass collected with IOM and CFC was 4.19 (95% CI = 3.79–4.64).	CFC/IOM inhalable aerosol mass: 4.2. CFC/IOM to inhalable aerosol mass for water-soluble: 1.6.	[42]
		25-mm CFC (‘total’)				
		IOM sampler (inhalable)				
Bronze foundry, copper ore smelter, lead-acid battery recycling, lead ore concentrate mill, solder manufacturing	Total and inhalable	IOM sampler (inhalable)	Lead	Bronze foundry: The filter sample size distributions did not differ significantly for IOM and CFC samplers (*p* < 0.05); Copper ore smelter: Only 1/7 pairs of IOM and CFC filters had a difference in mass-weighted size distributions; Lead acid battery recycling: Difference in one filter and wall distribution and one difference between IOM and CFC filter deposits; Lead ore concentrate mill: No filter sample differed significantly from wall deposit samples; Solder manufacturing: One IOM filter sample differed significantly from the corresponding CFC filter sample.	N/A	[31]
		37-mm plastic CFC (‘total’)				
Casting factory	Inhalable, respirable, thoracic	Novel three-stage sampler	Lead	Inhalable fraction: Three-stage sampler sampled lower corrections than the Respicon; Respirable fraction: The three-stage sampler and the IOSH cyclones collected similar respirable dust concentrations (less than 10% difference); Thoracic fraction: the three-stage sampler sampled lower concentrations than the Respicon (~22%).	1.5 for extra thoracic from inhalable Respicon (defined from other studies).	[41]
		IOSH cyclone				
		Respicon				
Copper electrorefinary	Inhalable	Disposable inhalable aerosol sampler	Copper	GM ratio of exposure measurements (DIAS/IOM) was 1.1 for the personal exposures, 1.6 for the area exposures, and 1.4 for the combined personal and area exposures. The DIAS sampled higher concentrations than the IOM sampler.	DIOS/IOM: 1.4	[45]
		IOM sampler				
Manufacture of indium-tin oxide	Inhalable and respirable	IOM sampler (inhalable)	Indium and dust	Inhalable: High agreement between dual IOM sampler and IOM sampler but lower agreement for dust (concordance correlation coefficient 0.997 vs. 0.886) Respirable: Better agreement for respirable indium compared to respirable dust (concordance correlation coefficient 0.932 vs. 0.777). Dual IOM sampler sampled higher respirable indium levels than the GK2.69 cyclone (1.61 mg/m^3^ mean ratio for IOM:Cyclone).	N/A	[29]
		Dual IOM sampler (inhalable and respirable)				
		GK2.69 cyclone (respirable)				
Manufacture of solder	Inhalable	IOM sampler	Lead	Only one filter sample from a corresponding wall deposit sample and one IOM filter sample significantly differed from the corresponding CFC filter sample.	N/A	[31]
		37-mm CFC				
	Total	37-mm CFC (filter only and wiping internal surfaces of cassette)	Lead and tin	No statistically significant differences for log transformed metal concentrations between filter/interior wipe (FW) samples and CI (*p* = 0.3009 for leas, *p* = 0.800 for tin). Significant differences between FO and CI results (*p* < 0.05).	N/A	[49]
		CFC which also includes a customised insert sample				
Magnesium foundry	Total, inhalable and respirable	IOM sampler (inhalable)	Beryllium	The median of median dust concentration ratios (no further discussion of median of median in the article) computed from the sampling heads at the fixed station decreased as follows: IOM (1.00) > Sierra (0.76) > 37-mm cassette (0.61) > MOUDI (0.48) > respirable (0.12). The median of median ratios of dust were as follows: (IOM (1.00) > Sierra (0.56) > 37-mm cassette (0.35) > respirable (0.06)) and Be (IOM (1.00) > Sierra (0.66) > 37-mm cassette (0.48) > respirable (0.11).	N/A	[39]
		37-mm CFC (‘total’)				
		SKC Aluminium cyclone (respirable)				
		8-stage Sierra cascade impactor				
		10-stage MOUDI impactors				
Manufacture of lead (lead powder factory and lead acid battery plant)	Inhalable, respirable, thoracic	Novel three-stage sampler	Lead	Inhalable fraction: Three-stage sample sampled lower corrections than the Respicon; Respirable fraction: The three-stage sampler and the IOSH cyclones collected similar respirable dust concentrations (less than 10% difference) Thoracic fraction: The three-stage sampler sampled lower concentrations than the Respicon (~22%).	1.5 for extra thoracic from inhalable Respicon (defined from other studies).	[41]
		IOSH cyclone				
		Respicon				
Metal smelter and metal foundry	Inhalable and respirable	IOM dual sampler	Metal	Inhalable mean concentrations: No statistically significant differences between samplers for inhalable fractions. Respirable concentrations: No statistically significant differences between samplers. IOM dual sampler sampled the highest concentrations (114–115%).	Not considered for field tests.	[28]
		Button sampler (inhalable)				
		Two-stage cascade impactor (inhalable and respirable)				
Processing of copper-beryllium	Total and inhalable	37-mm CFC (‘total’ and inhalable)	Beryllium	Personal samples GMs: CFC (5.88 ng m^−3^) and GSP (18.55 ng m^−3^) Static samplers GMs: CFC (7.41 ng m^−3^), GSP (18.55 ng m^−3^) and Respicon (respirable: 2.81 ng m^−3^, thoracic (5.44 ng m^−3^ and inhalable 8.03 ng m^−3^)	GSP/CFC: 2.88 for personal samples; 1.99 for static samples.	[36]
		GSP (‘total’ and inhalable)				
		Respicon				
**Other Sectors**						
Agriculture	Inhalable and respirable	Prototype sampler (inhalable)	General	The inhalable dust concentrations were approximately five times than that for respirable (*p* < 0.001, paired *t*-test). High correlation between IOM sampler and prototype sampler for inhalable mass concentrations (Spearman correlation coefficient = 0.85) with a mean difference of 0.03 mg/m^3^.	IOM/prototype ratio: 0.87.	[30]
		IOM sampler (inhalable)				
		GK2.69 cyclone (respirable)				
	Inhalable and respirable	IOM sampler (inhalable)	Agriculture	CFC/IOM GM (GSD) sampler ratios: Swine: 0.50 (1.2), chicken: 0.67 (1.5), turkey: 0.60 (1.3) and dairy: 0.49 (2.7); Button/IOM GM (GSD) ratios: Swine: 0.57 (1.6), chicken: 0.80 (1.4), turkey: 0.53 (1.3) and dairy: 0.69 (1.3); Cyclone/IOM GM (GSD) ratios: Swine: 0.05 (2.0), chicken: 0.08 (2.3), turkey 0.12 (1.7) and dairy 0.22 (3.8).	Button/IOM: Swine (0.57), chicken (0.80), turkey (0.53) and dairy (0.67). Conversion factor not recommended between cyclone and inhalable samplers.	[43]
		Button sampler (inhalable)				
		37-mm CFC (inhalable)				
		SKC Aluminium cyclone (respirable)				
Brick manufacturing	Inhalable and respirable	Higgins-Dewell cyclone (respirable)	Brick	For clay particulates: GM (GSD) of 0.75 (2.94) for Higgins-Dewell and 0.39 (2.60) for IOM; For mixed: GM (GSD) of 0.75 (2.63) for Higgins-Dewell and 0.36 (2.43) for IOM; For sand particles: GM (GSD) of 0.73 (4.29) for Higgins-Dewell and 0.46 (2.52) for IOM.	IOM dual fraction/cyclone: 0.50 clay particulates, 0.61 mixed and 0.96 for sand particles.	[37]
		IOM dual-fraction sampler (inhalable and respirable)				
Cement plants	Total, inhalable, thoracic, respirable	Higgins-Dewell cyclone (respirable)	Cement	Median ratio between thoracic aerosol and ‘total’ dust to be 0.41. The median ratios between observed results of the respirable, ‘total’ dust, and inhalable fractions relative to the thoracic aerosol fractions were 0.51, 2.4, and 5.9, respectively.	N/A	[38]
		GK2.69 cyclone (thoracic)				
		IOM sampler (inhalable)				
		37-mm CFC (‘total’)				
Road paving	Total and inhalable	37-mm CFC (‘total’)	Asphalt fumes	1:1 ratio between the two samplers when only limited to asphalt fumes. When there are confounders (dust or old asphalt fumes), significant differences were seen between the two samplers.	IOM/CFC: 1.37 for milling asphalt, 1.41 for asphalt paving over granular base, 1.02 for asphalt over asphalt pavements.	[44]
		IOM sampler (inhalable)				
	Total and inhalable	37-mm CFC	Fume sampling	The 37-mm CFC and the IOM sampler provided similar results with no statistical differences for collecting field organic aerosols from bitumen fumes.	IOM/CFC GM ratios: Mineral fraction: 1.82 Benzene-Soluble fraction: 1.02 Vapor fraction: 0.96.	[47]
		IOM sampler				
Rubber manufacturing	Inhalable	Seven hole sampler	Rubber dust	All the samplers apart from the IOM sampler under sampled the inhalable fraction.	GM (GSD) of samplers relative to CALTOOL: Seven hole sampler: 0.68 (1.41) IOM: 1.04 (1.41) 25-mm Millipore: 0.65 (1.66) 37-mm Millipore: 0.67 (1.50) PAS6-left: 0.73 (1.52) Polish sampler: 0.54 (1.69).	[46]
		PAS-6				
		25-mm Millipore				
		37-mm Millipore				
		IOM sampler				
		‘Polish’ sampler				
		Respicon (PSD)				
Shipbuilding	Total	37-mm CFC	Manganese	Significant correlations for measured concentrations between the two samplers (r = 0.964, *p* < 0.001).	N/A	[48]
		8-stage cascade impactor samplers				
Talc production plant, and a peat-fired power plant (mineral, metal, peat)	Inhalable and respirable	IOM dual sampler (inhalable and respirable)	Mineral, metal, peat	Inhalable: Mean concentration of inhalable dust of 2.5 mg/m^3^ (0.5–5.1 mg/m^3^) for the talc production plant and 42 mg/m^3^ (18–96 mg/m^3^) for the power plant. Respirable: Mean concentration of 0.2 mg/m^3^ (<0.1–0.3 mg/m^3^) for the talc production plant and 1.0 mg/m^3^ (<0.1–3.7 mg/m^3^) for the power plant.	N/A	[28]
		Button sampler (inhalable)				
		Two-stage cascade impactor (inhalable and respirable)				
Wood industry	Inhalable	IOM sampler	Wood	No statistically differences between the samplers for the measured woodworking processes. The Italian cone samplers and IOM sampler were exchangeable (*p* > 0.05).	Italian cone/IOM: 0.68 (not passive); 0.74 (when IOM sampler operated as a passive sampler).	[34]
		Italian cone sampler				
	Total and inhalable	37-mm CFC (‘total’)	Wood	For particles > 100 µm AED: Button versus IOM (*p* = 0.1781), CFC versus IOM (*p* = 0.1241) and Button versus CFC contrast is not significant (*p* = 0.999). For samples without particles of 100 µm or larger: Button versus CFC contrast is significant (*p* = 0.04). Button versus IOM contrast is significant (*p* = 0.005).	Discussed in the article but are not applied to the results.	[32]
		IOM sampler (inhalable)				
		Button sampler (inhalable)				
	Total and inhalable	37-mm CFC (‘total’)	Wood	The samplers were not significantly different for measured concentrations compared to CALTOOL. The CFC sampler collected the least dust compared to the other samplers with the IOM sampler collecting two times more dust than the CFC.	Ratio R: sampler concentration/CALTOOL mouth mean concentrations: IOM 1.12, CIP 0.94, ACCU-CAP 0.8, Button 0.86, CFC 0.62.	[33]
		IOM sampler (inhalable)				
		CIP 10-l (inhalable)				
		ACCUCAP (inhalable)				
		Button sampler (inhalable)				
	Inhalable	37-mm CFC with ACCU-CAP	Wood	Median dust concentrations of 0.90 for ACCU-CAP, 0.87 for the Button sampler, 1.08 for CIP-10l, 0.95 for the GSP and 1.06 mg/m^3^ for the IOM sampler.	ACCU—CAP/ACCU-CAP: 0.16 ± 0.50; ACCU-CAP/Button: 0.91 ± 0.46; ACCU-CAP/CIP10-l: 0.60 ± 1.52; ACCU-CAP/GSP: 0.77 ± 0.75; ACCU-CAP/IOM: 0.64 ± 0.38; Button/Button: 0.97 ± 0.34; Button/CIP10-l: 0.82 ± 1.18; Button/GSP: 0.79 ± 0.57; Button/IOM: 0.95 ± 0.52; CIP-10l/CIP10-l: 1.13 ± 1.02; CIP10-l/GSP: 1.08 ± 0.35; CIP10-l/IOM: 1.08 ± 0.39; GSP-GSP: 0.88 ± 0.26 GSP/IOM: 0.99 ± 0.39; IOM/IOM: 0.74 ± 0.74.	[35]
		Button				
		CIP-10l				
		GSP				
		IOM sampler				
	Inhalable, respirable and thoracic	Respicon (inhalable, respirable, thoracic)	Wood	Inhalable: No significant difference between IOM sampler and Respicon when a correction factor for the Respicon was applied. The Respicon under sampled by 23% when no correction factor was applied. Respirable: Significant difference between the Respicon and the SKC cyclone. Thoracic: Respicon oversampled the extrathoracic dust fraction resulting in an overall error of 48%.	Respicon correction factor of 1.5 for extrathoracic (from previous studies).	[40]
		IOM plastic sampler (inhalable)				
		SKC Aluminum cyclone (respirable)				
		GK 2.69 cyclone (thoracic)				

### 3.4. EN Standard 13205:5 for Workplace Comparison Studies

Table 4 summarises the requirements of the standard and if these are considered in the article. It is clear that the standard requirements are not being followed. In most cases, it is not possible to evaluate if the requirements of the standard have been met due to limited information provided in the article for the sampling measurements. This is particularly the case for a number of aspects of the standard:

- Number of experiments. The standard requires that four sets of experiments (consisting of five runs and parallel sampling) are performed. From the studies, it is unclear if this is being followed.
- Candidate sampler bias. For the standard a minimum of five different experimental runs for validated sampler/candidate sampler are required to be used. From the studies, it is unclear if this is being followed.
- Sampler bias and expanded uncertainty. In the table, those studies which have reported the correction factors (either calculated or stated) have been assigned as partially meeting the standard. However, no studies clearly meet this requirement of the standard.

## 4. Discussion

### 4.1. Samplers Assessed

This present article provides a review of workplace sampler comparison studies available in the peer-reviewed literature published between 2004–2020.

The most common particulate fraction assessed is the inhalable fraction in more than two third of the articles. This finding is expectant. The inhalation fraction is the primary aerosol fraction of interest. This is primarily due to OELVs, threshold limit values (TLV) and other limit values which primarily refer to the inhalable fraction. For example, the limit values for lead and inorganic compounds including in the EU, Canada, Japan, China, South Korea, and the USA are based on the inhalable fraction [8]. The most common inhalable sampler studied is the IOM inhalable sampler (56%) followed by the Button sampler. The IOM sampler is most frequently considered to be the gold standard sampler for the inhalable fraction [9].

A third of the articles compared samplers for the respirable fraction with cyclones (such as the SKC Aluminium cyclone) used. There has also been an increasing interest for assessing the respirable fraction in the metals industry, based on specific toxicological endpoints [6,52,53]. It is therefore important that a good body of evidence is available for how the different respirable samplers compare with each other for measurements in the metals industry. Further work to expand this evidence for respirable samplers in the metals industry is required.

The only thoracic only sampler used has been the GK2.69 cyclone. In approximately 40% of the articles, the total fraction has been assessed, with the 37-mm CFC most commonly used. Some samplers have only been used in one article which does not allow the sampler results to be cross-referenced with other articles.

**Table 4:** Comparison of studies with requirements of EN 13205:5 standard.

Reference	Reference to EN 13205 Standard	Number of Experiments (as Stated in the Standard)	Candidate Sampler Bias	Candidate Sampler Variability	Exclusion from the Nominal Flow Rate	Collected Mass or Internally Separated Mass	Sampler Bias and Expanded Uncertainty
[28]	x	U	U		N/A (inhalable) x (respirable)	x	x
[29]	x	U	U	x	N/A (inhalable) x (respirable)	x	x
[30]	x	U	U	x	N/A (inhalable) x (respirable)	x	P
[31]	x	U	U	N/A	N/A	x	P
[32]	x	U	U	x	N/A (inhalable) x (total)	x	x
[33]	x	U	U	N/A	N/A	x	x
[34]	x	U	U	N/A	N/A	x	P
[35]	x	U	U	N/A	N/A	x	P
[36]	x	U	U	x	N/A (inhalable) x (total)	x	P
[37]	x	U	U	x	N/A (inhalable) x (respirable)	x	P
[38]	x	U	U	x	N/A (inhalable) x (total, thoracic, respirable)	x	x
[39]	x	U	U	x	N/A (inhalable) x (respirable, total)	x	x
[40]	x	U	U	x	N/A (inhalable) x (respirable, thoracic)	x	P
[41]	x	U	U	x	N/A (inhalable) x (respirable, thoracic)	x	P
[42]	x	U	U	x	N/A (inhalable) x (total)	x	P
[43]	x	U	U	x	N/A (inhalable) x (respirable)	x	P
[44]	x	U	U	x	N/A (inhalable) x (total)	x	P
[45]	x	U	U	N/A	N/A	x	P
[46]	√	U	U	N/A	N/A	x	x
[47]	x	U	U	x	N/A (inhalable) x (total)	x	P
[48]	x	U	U	x	x (total)	x	x
[49]	x	U	U	x	x	x	x

Key: U: unclear if meets the requirement. P: partly meets the requirement. x: does not meet the requirement. N/A: the requirement is not applicable.

### 4.2. Comparison of Samplers Reported in the Literature and Those Used by Industry

CEN TR 15230 [54] gives a very good overview of the available sampling techniques used 15 years ago complying with the inhalable, thoracic, and respirable fractions as defined in the earlier mentioned standards. Since then, limit values for metals and metalloids have been proposed and set following those conventional fractions, new sampling systems came on the market and for many there are no comparison studies available nor is it clear whether they all meet the sampling efficiency requirements. When comparing the samplers reported in the literature and those used within the metals industry (personal communication, S Verpaele (Appendix A), a large number of samplers have not been identified in literature for sampler comparison studies. No relevant sampler comparison studies were identified in our searches for the following samplers:

- Inhalable samplers: Open face cassette (OFC) 25 mm, Zefon inhalable sampler, HSE 7H sampler, and the 37 mm conical inhalable sampler (CIS);
- Respirable samplers: FSP2, SKC conductive plastic cyclone, Zefon cyclones. GS-1 and GS-3 respirable cyclones, SKC disposable, and aluminium respirable PPI samplers, and the 10 mm Dorr-Oliver Nylon Cyclone;
- Multifraction samplers: EA sampling system;
- PM fractions: SKC personal environment monitors for PM2.5 and PM10, SKC PM2.5, and PM10 IMPACT samplers, SKC PM coarse IMPACT sampler and SKC personal modular impactor (PMI) samplers for PM2.5 and PM10.

This review is also limited in timescales (2004–2020), so it may be the case that some published studies on these samplers are potentially available. Additional complementary searches were performed for these samplers to identify potentially relevant sampler comparison studies for these samplers pre-2004 in PubMed. No relevant studies were easily identified. This is a key data gap in the literature and illustrates the requirement of an international sampler comparison study to be undertaken (discussed in Section 4.6).

### 4.3. EN Standard 13025:5 for Workplace Sampler Comparison Studies

The requirements for workplace sampler comparison in EN13025:5 for workplace sampler performance should be followed and for the majority of the studies considered in this review the requirements of this standard were not discussed (or standard even referred to). Authors should be referring to these standards for performing workplace sampler comparison studies and ensuring the appropriate experiments are being performed and recorded. In fact, only one article [46] refers to the standard.

Using the standard will also allow the generation of correction factors which can then be applied to data. This will also allow data to be pooled and also used for comparison purposes.

### 4.4. Workplace Settings

Metal/metalloids settings comprised the dominant setting. Only aluminium smelters had two publications that studied exposure [39,42] with other metal settings only being subject to one publication. Nonmetal/metalloids settings were limited to nine publications. With the exception of agriculture (two studies; [30,43]) and wood settings (five studies; [32,33,34,35,40], settings with one publication included brick manufacturing (for brick dust) and road paving.

In agriculture settings the differences between the samplers was insignificant [30]. In brick manufacturing, one aspect that needs to be considered is the type of dust that is being sampled [37]. The respirable fraction was sampled in four identified articles with the thoracic fractions assessed in six articles where the Respicon sampler was used in 50% of these articles.

Only a limited number of metal dusts have been assessed (six in total—aluminium, beryllium, copper, lead, tin, and general metal) which suggests a potential research gap for assessing the most appropriate sampler to be used for measuring other metal dusts. For beryllium dust, one article has recommended that the use of inhalable measurements for sampling over the respirable and fractions is recommended until a dose–response curve has been undertaken for the sampler heads for the respirable and thoracic fractions [39]. Oversampling and under sampling are potential aspects that has been highlighted for sampling dusts, for example for lead, the respirable fraction has been overestimated by the IOSH cyclone [41]. In the case of under sampling, a number of samplers underestimated rubber dust [46]. Sampling location can also affect the sampler comparison which was observed by Lee et al. [49] for copper dust, where there was statistically significant differences between the area measurements but not for personal measurements. In workplace settings, measurements in wood environments have been undertaken allowing a greater sampler comparison to be undertaken. In a number of cases, the samplers produced similar results such as the ACCU-CAP, Button, CIP10-L, GSP, and IOM samplers [35]. This was also the case for the Italian Cone sampler and the IOM sampler for the inhalable fraction for wood dust [34].

For correction factors reported (Table 3), no trends could be identified between samplers and settings which further illustrates the need to follow the standard so that correction factors are performed in a similar manner.

### 4.5. Limitations Identified by Article Authors

A number of limitations have been identified by article authors. Limitations with foam insert have been identified in two studies. The foam insert has been identified as a potential limitation by De Vocht et al. [37] where clay particulates could stick to the insert or together. Lee et al. [49] also identified that using a customized insert in a cellulosic sampler may not be appropriate for gravimetric analysis due to changes in humidity and mass variabilities. Potential errors from the wiping process of the samplers has been highlighted by Lee et al. [45] such as an inconsistent pressure being applied for wiping the cap inside and internal walls. The sampler location has been noted by Lee et al. [35] as a potential issue with one sampler (CFC) moving position during the sampling process, from the opener facing a 45 degree angle to the vertical at the beginning to being observed to be pointing face outward from the body at the end of the sampling. Potential under sampling has been highlighted by Campopiano et al. [34]. Only the particles on the sampling substrate were determined by Campopiano et al. [34] with the wall deposition not considered for the IOM cassette.

### 4.6. Potential Improvements

From this review, a number of aspects need to be considered by both article authors and the reviewers of the articles as part of the peer-reviewing process. The first aspect concerns the EN 13205 standard. It is clear from this review that the requirements of the standard are not being met as suggested by the contents of the peer-reviewed publication. Particularly, in the cases for the number of experiments and sampler bias, it is not clear in the article if these requirements are being met.

Following on from the collected mass or internally separated mass requirement of EN13205, the contribution of wall deposits needs to be discussed by authors. The contribution of wall deposits has only been considered in a limited number of studies. The inclusion of wall deposits has been taken into account for the sampled mass in studies such as Lee et al. [45] where the internal walls were wiped and the mass of the wipes include and Anthony et al. [30], where an internal capsule was used to collect wall deposits for a prototype sampler. A number of studies have mentioned wall deposits; however, they have not included the contribution of wall deposits in the results [36,39] A number of studies include no discussion on wall deposits. The issue of dealing wall losses (where the particle is not collected on the filter but is deposited on other parts of the sampler inlet) is an ongoing challenge [5].

It is considered that more detailed information is required for the samplers to be included by article authors, and if not included, requested by reviewers. This includes cassette materials, for example in the case of the IOM sampler, where in some cases it is not stated if a plastic or stainless-steel cassette has been used in the article. Flow rates also need to be included for the samplers used, even if only the recommended flow rates for the samplers are used.

Other areas identified which need to be considered are handling of cassettes and wiping of cassettes (where appropriate), handling and storage of filter materials and sampling measurements, and duration need to be explicitly clear.

Developed samplers should also be named to allow data tracking for the sampler to occur. This was the case for one sampler that was developed by L’Orange et al. [50] which has subsequently been tested by Anthony et al. [30] using a different name (prototype high-flow inhalable dust sampler).

These suggested improvements reinforce that comprehensive data sharing should be encouraged by authors and requested by reviewers, for example through the use of supplementary material. The limitations identified and suggested improvements can be overcome by this comprehensive data sharing which will allow better communication within the community. This communication can also allow technology improvements for workplace sampling.

## 5. Conclusions

A narrative review has been performed for workplace personal sampler comparison studies between 2004–2020, with 22 studies identified as being relevant from defined inclusion and exclusion criteria. The majority of studies assessed the inhalable fraction (with the IOM inhalable sampler being the most assessed sampler), followed by the respirable fraction with only one study identified for the thoracic fraction. The most common setting for the sampler comparison settings has been in metal/metalloids settings. A number of personal samplers used in industry not being the subject of sampler comparison studies in the literature such as the Zefon inhalable sampler and SKC disposable respirable PPI samplers for the respirable fraction.

A number of the included studies have identified limitations and data gaps and across the studies, the need for additional information has been highlighted. This includes the need to provide full information on, for example, the samplers tested, handling of cassettes, filters, etc., cleaning protocols, detailed analysis information, and full information on standards that have been used for assessment.

EN-13205 should be followed for performing workplace sampler comparison studies. It is evident from the literature, this standard is not being followed, or certainly, the available published information suggests that this is the case. Following EN13205 will also allow correction factors to be assessed in a similar manner, allowing comparison between samplers in different settings.

It is evident that there is need for developed standard operating procedures, which follow the requirements of EN-13205, which can be followed by both researchers, journal article authors, and journal reviewers. Developing a clear standardized protocol for performing comparison and performance studies for use is one potential approach for the way forward. This is currently being investigated as part of the SCSG. The SCSG invite stakeholders in the scientific and industrial community to be involved in these discussions. For further information, please contact Steven Verpaele (sverpaele@nickelinstitute.org).

## Figures and Tables

**Table 1:** Selected Experimental Requirements for EN13025:5 for workplace sampler performance [27].

Parameter	Description
Number of experiments	The standard requires that four sets of experiments (consisting of five runs and parallel sampling) are performed. The parallel runs set out in the standard to be performed are between the candidate and validated samplers, for a least six candidate specimens for the personal sampler, the determination of the effect of flow rate excursions on the mass fraction samples, and the determination of internally separated mass or the collected mass effect. It is also required, under the standard that a validated sampler is needed to be used for two of the experiments.
Candidate sampler bias	For complying with the standard, the exclusion of outliers is allowed. However, a minimum of five different experimental runs for validated sampler/candidate sampler are required to be used.
Candidate sampler variability (not applicable for inhalable fraction and large static samplers)	The standard discusses that this test is not necessary if the candidate sampler is for personal inhalable sampling or if the candidate sampler is a large static sampler.
Exclusion from the nominal flow rate (not applicable for inhalable fractions)	The standard sets out the requirements for sampling the respirable and thoracic fraction. This involves calculating the corresponding uncertainty component. This requirement of the standard is not applicable for sampling the personal inhalable fraction.
Collected mass or internally separated mass	The standard discusses that the tests required for collecting the mass and internally separated mass can be performed simultaneously. If the inhalable fraction is being sampled, it is stated that the second test is not required. The components to be calculated are the maximum collected mass and the maximum internally separated mass.
Sampler bias and expanded uncertainty	The criterion for applying a correction factor is stated in the standard for a candidate sampler to be validated. The correction factor from the manufacturer can be used or a correction factor obtained from a relevant measuring procedure. In cases of no correction factor being stated, the standard stated a value of 1.00 should be used.

## Data Availability

The data presented in this study are available within the article and its Supplementary Material (Appendix A, Appendix B and Appendix C). Details of the references for which the data was extracted are provided in the reference list.

## Appendix A

**Table A1:** List of samplers identified as being used by the metals industries (personal communication, S Verpaele).

Inhalable	Thoracic	Respirable	Multi-Fraction	Other Fraction Samplers
Open face cassette (OFC) 25 mm	SKC Disposable Thoracic Parallel Particle Impactors (PPI^®^) (blue) 2 L min^−1^	FSP 2	EA sampling system	SKC Personal Environmental Monitor (PEM) (blue) for Particulate Matter (PM) 2.5 (2.5 µm) 2 L min^−1^
Closed face cassette (OFC) 37 mm	SKC Aluminium Thoracic PPI (blue) 2 L min^−1^	FSP 10	RESPICON	SKC PEM (black) for PM2.5 (2.5 µm) 4 L min^−1^
Closed face cassette (CFC) 25 mm		SKC conductive plastic cyclone		SKC PEM (red) for PM2.5 (2.5 µm) 10 L min^−1^
Closed face cassette (CFC) 37 mm		10 mm Dorr-Oliver Nylon Cyclone		SKC PEM (green) for PM10 (10 µm) 2 L min^−1^
GSP 3.5		Zefon 37 mm Aluminium Cyclone		SKC PEM (gold) for PM10 (10 µm) 4 L min^−1^
GSP 10		Zefon 25 mm Aluminium Cyclone		SKC PEM (bronze) for PM10 (10 µm) 10 L min^−1^
Zefon inhalable dust sampler, reusable, plastic		Zefon 10 mm Conductive Nylon Cyclone		SKC PM2.5 IMPACT Sampler
Zefon Disposable Inhalable Sampler (DIS) with Cellulose Filter Capsule		GS-1 Respirable Cyclone		SKC PM10 IMPACT Sampler
Zefon DIS with Polyvinyl chloride (PVC) Filter Capsule		GS-3 Respirable Dust Cyclone		SKC PM Coarse IMPACT Sampler
Button Aerosol Sampler		SKC Aluminium Cyclone		SKC Personal Modular Impactor (PMI) (gold) PM2.5
IOM Sampler and Cassette, Plastic		SKC Disposable PPI^®^ (gold) 2 L min^−1^		SKC PMI (silver) PM10
IOM Inhalable Sampler and Cassette, Stainless Steel		SKC Disposable Respirable PPI^®^ (orange) 4 L min^−1^		SKC PMI (gold/silver) PM Coarse
Health and Safety Executive (HSE) 7H sampler		SKC Disposable Respirable PPI^®^ (red) 8 L min^−1^		SKC PEM (blue) for PM2.5 (2.5 µm) 2 L min^−1^
PAS-6		SKC Aluminium Respirable PPI (gold) 2 L min^−1^		SKC PEM (black) for PM2.5 (2.5 µm) 4 L min^−1^
37 mm Conical Inhalable Sampler (CIS)		SKC Aluminium Respirable PPI (orange) 4 L min^−1^		SKC PEM (red) for PM2.5 (2.5 µm) 10 L min^−1^
Swinnex sampling head (13 mm)		SKC Aluminium Respirable PPI (red) 8 L min^−1^		

## Appendix B

**Table A2:** Workplace based sampler comparison article settings.

Setting	Aerosol Fraction Assessed/Compared	Samplers	Cassette Materials	Sampling Substrate Material	Type of Sampling	Flow Rate (L/min)	Particle Size Distribution (PSD) Considered?	Reference
**Metals Sector**								
Aluminium smelters	Total, inhalable, respirable and PSD	IOM inhalable sampler	Not stated	25-mm MCE; pore size 0.8 µm	Personal and area	2	Yes	[39]
		SKC Aluminium cyclone	Aluminium	37-mm MCE; pore size 0.8 µm		2.5		
		37-mm CFC	Plastic	MCE; pore size 0.8 µm		2.5		
		8-stage Sierra cascade impactor	Not stated	34-mm MCE (pore size 0.52 µm–21.3 µm fractions)		Not stated		
		10-stage MOUDI impactors	Not stated	37-mm MCE (pore size 0.056 µm–21.3 µm)		30		
	Total and inhalable	Respicon	Not stated	PVC (pore size 5 µm)	Personal	3.1	No	[42]
		25-mm CFC	Not stated	PVC (pore size 5 µm)		2		
		IOM sampler	Not stated	Cellulose ester (pore size 5 µm)		2		
Bronze foundry, copper smelter, lead-acid battery recycling, solder manufacturing	Total and inhalable	IOM sampler	Stainless steel	MCE (pore size 0.8 µm)	Area	2	No	[31]
		37-mm plastic CFC	Plastic			2		
Casting factory	Inhalable, respirable, thoracic	Novel three-stage sampler	Not stated	Glass; pore size not discussed	Area	3.2	No	[41]
		IOSH cyclone	Not stated	Not discussed		Not stated		
		Respicon	Not stated	Not discussed		Not stated		
Copper electrorefinary	Inhalable	Disposable inhalable aerosol sampler (DIAS)	Not stated	37-mm MCE; pore size 0.8 µm	Personal and area	2	No	[45]
		IOM sampler	Not stated	25-mm MCE; pore size 0.8 µm		2		
Manufacture of indium-tin oxide	Inhalable and respirable	IOM sampler	Plastic	PVC; pore size 5-mm	Area	2	No	[29]
		Dual IOM sampler	Plastic	PVC; pore size 5-mm		2		
		GK2.69 cyclone	Not stated	PVC; pore size 5-mm		4.2		
Manufacture of solder	Inhalable	IOM sampler	Stainless steel	Standard MCE; pore size 0.8 µm	Area	2	No	[31]
		37-mm CFC	Plastic			2		
	Total	37-mm CFC (filter only and wiping internal surfaces of cassette)	Not stated	37-mm MCE; pore size 0.8 µm	Personal and area	2	No	[49]
		CFC which also includes a customised insert sample	Not stated			2		
Magnesium foundry	Total, inhalable, and respirable and PSD	IOM sampler	Not stated	25-mm MCE; pore size 0.8 µm	Personal and area	2	Yes	[39]
		37-mm CFC	Aluminium	MCE; pore size 0.8 µm		2.5		
		SKC Aluminium cyclone	Plastic	MCE; pore size 0.8 µm		2.5		
		8-stage Sierra cascade impactor	Not stated	34-mm MCE; pore size 0.52 µm–21.3 µm fractions)		Not stated		
		10-stage MOUDI impactors	Not stated	37-mm MCE; pore size 0.056 µm–18 µm fractions)		30		
Manufacture of lead (lead powder factory and lead acid battery plant)	Inhalable, respirable, thoracic	Novel three-stage sampler	Not stated	Glass; pore size not discussed	Area	3.2	No	[41]
		IOSH cyclone	Not stated	Not discussed		Not stated		
		Respicon	Not stated	Not discussed		Not stated		
Metal smelter and metal foundry	Inhalable and respirable	IOM head with porous plastic foam insert	Stainless steel (dual IOM)	25 mm cellulose acetate; pore size not discussed	Area	2	No	[28]
		Button sampler	Not stated			2		
		Two-stage cascade impactor	Not stated	47-mm Teflon; pore size not discussed		10		
Processing of copper-beryllium	Total and inhalable	37-mm CFC	Not stated	MCE; pore size 5 µm	Personal and area	2	No	[36]
		GSP	Not stated			3.5		
		Respicon	Not stated			3.1		
**Other Sectors**								
Agriculture	Inhalable and respirable	Prototype sampler	Not stated	PTFE; pore size 2 µm	Area	2	No	[30]
		IOM sampler	Not stated	PVC; pore size 5 µm		2		
		GK2.69 cyclone	Not stated	PVC; pore size 5 µm		2		
	Inhalable and respirable	IOM sampler	Plastic	PVC; pore size 5 µm	Area	2	No	[43]
		Button sampler	Not stated			4		
		37-mm CFC	Not stated			2		
		SKC Aluminium cyclone	Aluminium			2.5		
Brick manufacturing	Inhalable and respirable	Higgins-Dewell cyclone	Plastic	37-mm PVC membrane; pore size 5.0 mm	Area	2.2	No	[37]
		IOM dual-fraction sampler	Plastic	25-mm PVC membrane; pore size 5.0 mm		2		
Cement plants	Total, inhalable, thoracic, respirable	Higgins-Dewell cyclone	Not stated	37-mm PVC; pore size not discussed	Personal	2.2	No	[38]
		GK2.69 cyclone	Cellulose support	37-mm PVC; pore size not discussed		1.6		
		IOM sampler	Plastic	25-mm PVC; pore size not discussed		2		
		37-mm CFC	Not stated	37-mm PVC; pore size not discussed		2		
Road paving	Total and inhalable	37-mm CFC	Not stated	Glass fibre; pore size not discussed	Personal	Not given	No	[44]
		IOM sampler	Stainless steel	25-mm glass fibre; pore size not discussed		Not given		
	Total and inhalable	37-mm CFC	Not stated	PTFE; pore size not discussed	Personal	Not given	No	[47]
		IOM sampler	Stainless steel	Fiberglass membrane; pore size not discussed		Not given		
Rubber manufacturing	Inhalable	Seven hole sampler	Not stated	Whatmann glass fibre filters, diameter depending on type of sampler (25 mm, 37 mm, 47 mm or 50 mm); pore size not discussed	Area	2	Yes	[46]
		PAS-6				2		
		25-mm Millipore	Not stated			2		
		37-mm Millipore	Not stated			2		
		IOM sampler	Not stated			2		
		‘Polish’ sampler	Plastic			2		
		Respicon (PSD)	Not stated			2		
Shipbuilding	Total and PSD	37-mm CFC	Not stated	MCE; pore size not discussed	Personal	2	Yes	[48]
		8-stage cascade impactor samplers	Not stated			2		
Talc production plant, and a peat-fired power plant (mineral, metal, peat)	Inhalable and respirable	IOM dual sampler	Stainless steel	25-mm cellulose acetate; pore size not discussed	Area	2	No	[28]
		Button sampler	Not stated			2		
		Two-stage cascade impactor	Not stated	47-mm Teflon; pore size not discussed		10		
Wood industry	Inhalable	IOM sampler	Not stated	25-mm PVC; pore size 5 µm	Personal	2	No	[34]
		Italian cone sampler	Not stated			3.5		
	Total and inhalable	37-mm CFC	Not stated	MCE; pore size not discussed	Personal	Not given	No	[32]
		IOM sampler	Not stated			Not given		
		Button sampler	Not stated			Not given		
	Total and inhalable	37-mm CFC	Not stated	37-mm PVC; pore size 5 µm	Personal and area	1	No	[33]
		IOM sampler	Not stated	25-mm PVC; pore size 5 µm		2		
		CIP 10-l	Not stated	Foams housed in cups		10		
		ACCUCAP	Not stated	MCE; pore size 0.8 µm		2		
		Button sampler	Not stated	25-mm PVC; pore size 5 µm		1		
	Inhalable	37-mm CFC with ACCU-CAP	ACCU-CAP: PVC CFC: not stated	37-mm PVC; pore size 5 µm	Personal	2	No	[35]
		Button	Not stated	25-mm glass fibre; pore size not discussed		4		
		CIP-10l	Not stated	Polyurethane foam; pore size not discussed		10		
		GSP	Polyurethane	PVC; pore size not discussed		3.5		
		IOM sampler	Not stated	25-mm PVC; pore size not discussed		2		
	Inhalable, respirable and thoracic	Respicon		37-mm glass fibre	Area	3.1	No	[40]
		IOM plastic sampler	Plastic	25-mm Teflon; pore size 1 µm		2		
		SKC Aluminium cyclone	Aluminium	37-mm Teflon pore size; 1 µm		2.5		
		GK 2.69 cyclone	Aluminium	47-mm Teflon; pore size 1 µm		1.7		

Abbreviations: MCE: Mixed Cellulose Ester, PTFE: Polytetrafluoroethylene. PVC: Polyvinyl chloride.

## Appendix C

**Table A3:** Workplace based sampler comparison article settings.

Setting	Aerosol Fraction Assessed/Compared	Samplers	Dust Assessed	Number of Measurements	Analysis Undertaken	Analysis Details	Wall Loses and Deposits	Reference
**Metals Sector**								
Aluminium smelters	Total, inhalable, respirable	IOM inhalable sampler	Beryllium	68 dust samples (64 analysed for Be)	Gravimetric and chemical	Only the filter catch was analysed. No further explanation of catch in the article	Total mass (dust) not corrected for inside wall loses	[39]
		SKC Aluminium cyclone		57 dust samples (56 analysed for Be)				
		37-mm CFC		61 dust samples analysed for Be				
		8-stage Sierra cascade impactor		64 dust samples (54 analysed for Be)				
		10-stage MOUDI impactors		25 dust samples (19 analysed for Be)				
	Total and inhalable	Respicon	Aluminium	Total of 2275 full-shift air samples	Gravimetric and chemical	IOM cassettes and all filters weighed	Wall deposits collected for CFC and Respicon	[42]
		25-mm CFC						
		IOM sampler						
Bronze foundry, copper smelter, lead-acid battery recycling, solder manufacturing	Total and inhalable	IOM sampler	Lead	Bronze foundry: seven pairs; Solder manufacturing: five pairs; Lead ore concentrate mill: eight pairs and additional non-paired IOM and CFC sample	SEM and chemical	Filter used for ICP-MS analysis	Wall deposits analysed by SEM/EDX	[31]
		37-mm plastic CFC						
Casting factory	Inhalable, respirable, thoracic	Novel three-stage sampler	Lead	At least six effective samples for each workplace	Gravimetric	Not mentioned for field sampling	Not mentioned for field sampling	[41]
		IOSH cyclone						
		Respicon						
Copper electrorefinary	Inhalable	Disposable inhalable aerosol sampler (DIAS)	Copper	48 paired measurements (23 personal and 25 area)	Chemical	Internal walls wiped for analysis. Filter, internal wall wipe and cap wipe mases combined	Internal walls wiped for analysis	[45]
		IOM sampler	Not stated	25-mm MCE; pore size 0.8 µm				
Manufacture of indium-tin oxide	Inhalable and respirable	IOM sampler	Indium and dust	17 pairs of IOM (dual) and respirable cyclone samples; 18 pairs of IOM (dual) and IOM samples	Gravimetric and chemical; Inhalable: entire sampler and filter weighed; Respirable: bottom plastic support and filter weighed	IOM cassette wiped with wetted PVC filter	Not discussed	[29]
		Dual IOM sampler						
		GK2.69 cyclone						
Manufacture of solder	Inhalable	IOM sampler	Lead	Five pairs	SEM and chemical	Filter used for ICP-MS analysis	Wall deposits analysed by SEM/EDX	[31]
		37-mm CFC						
	Total	37-mm CFC (filter only and wiping internal surfaces of cassette)	Lead and tin	Personal samples: smelting—8 samples, casting—9 samples, powder handling—16 samples, others—6 samples Area samples: Smelting—8 samples, powder- 5 samples	Chemical	Complete dissolution of insert with acid and filter plus internal wipe for mass concentrations	Wall deposits were measured	[49]
		CFC which also includes a customised insert sample						
Magnesium foundry	Total, inhalable, and respirable and PSD	IOM sampler	Beryllium	68 dust samples (64 analysed for Be)	Gravimetric and chemical	Only the filter catch was analysed	Total mass (dust) not corrected for inside wall loses	[39]
		37-mm CFC		57 dust samples (56 analysed for Be)				
		SKC Aluminium cyclone		61 dust samples analysed for Be				
		8-stage Sierra cascade impactor		64 dust samples (54 analysed for Be)				
		10-stage MOUDI impactors		25 dust samples (19 analysed for Be)				
Manufacture of lead (lead powder factory and lead acid battery plant)	Inhalable, respirable, thoracic	Novel three-stage sampler	Lead	At least six effective samples for each workplace	Gravimetric	Not mentioned for field sampling	Not mentioned for field sampling	[41]
		IOSH cyclone						
		Respicon						
Metal smelter and metal foundry	Inhalable and respirable	IOM head with porous plastic foam insert	Metal	15 for metal smelter and foundry	Gravimetric	Whole cassettes weighed for IOM; only filter weighed for Button	Not discussed	[28]
		Button sampler						
		Two-stage cascade impactor						
Processing of copper-Beryllium	Total and inhalable	37-mm CFC	Beryllium	39 personal samples and 21 static samples taken	Chemical only	Only filters used for analysis	Wall loses were not considered for exposure concentrations	[36]
		GSP						
		Respicon						
**Other Sectors**								
Agriculture	Inhalable and respirable	Prototype sampler	Agriculture	36 paired samples for gravimetric analysis	Gravimetric	All sample media was weighed (×3 times)	Internal capsule to collect wall deposits	[30]
							Discussed sampler includes wall deposits	
		IOM sampler				Not discussed	Not included	
		GK2.69 cyclone						
	Inhalable and respirable	IOM sampler	Agriculture	20 pairs for field sampling	Gravimetric	Filters weighed	Not discussed	[43]
		Button sampler						
		37-mm CFC						
		SKC Aluminium cyclone						
Brick manufacturing	Inhalable and respirable	Higgins-Dewell cyclone	Brick	72 pairs	Gravimetric	Filters weighed	Not discussed	[37]
		IOM dual-fraction sampler						
Cement plants	Total, inhalable, thoracic, respirable	Higgins-Dewell cyclone	Cement	112 pairs for respirable/thoracic, 112 pairs for thoracic/inhalable and 122 pairs for respirable/inhalable; 72 sets available for all fractions	Gravimetric	Filters weighed	Not discussed	[38]
		GK2.69 cyclone						
		IOM sampler						
		37-mm CFC						
Road paving	Total and inhalable	37-mm CFC	Not stated Asphalt fumes	54 worker samples	Gravimetric and chemical	Cassette and filters weighed	Discussed that inner cassette wall deposits should be included in BEIP fraction	[44]
		IOM sampler						
	Total and inhalable	37-mm CFC	Fume sampling	Unclear- possibly 46 paired samples	Chemical	Extraction from filters	Not discussed	[47]
		IOM sampler						
Rubber manufacturing	Inhalable	Seven hole sampler	Rubber dust	281 measurements in total	Gravimetric	Filters only weighed	Not discussed	[46]
		PAS-6						
		25-mm Millipore						
		37-mm Millipore						
		IOM sampler						
		‘Polish’ sampler						
		Respicon (PSD)						
Shipbuilding	Total and PSD	37-mm CFC	Manganese	86 for each sampler	Gravimetric	Filter only	Not discussed	[48]
		8-stage cascade impactor samplers						
Talc production plant, and a peat-fired power plant (mineral, metal, peat)	Inhalable and respirable	IOM dual sampler	Mineral, metal, peat	21 for talc production plant, 15 for metal smelter and foundry and 10 for the power plant	Gravimetric	Foams and filter weighed	Not considered	[28]
		Button sampler						
		Two-stage cascade impactor						
Wood industry	Inhalable	IOM sampler	Wood	136 IOM/Italian cone samples and 136 passive IOM samples. 114 valid sample pairs	Gravimetric and SEM	Only filter weights used for analysis	Results does not include wall deposits from IOM cassette	[34]
		Italian cone sampler						
	Total and inhalable	37-mm CFC	Wood	62 for CFC, 59 for Button and 65 for IOM	Microscopy	Filter analysed	Inner wall deposits included for CFC	[32]
		IOM sampler						
		Button sampler						
	Total and inhalable	37-mm CFC	Wood	235 sample pairs	Gravimetric	Filters, IOM and ACCUCAP cassettes and CIP10-l cups weighed	Not discussed	[33]
		IOM sampler						
		CIP 10-l						
		ACCUCAP						
		Button sampler						
	Inhalable	37-mm CFC with ACCU-CAP	Wood	444 pairs	Gravimetric	Filters and foams weighed	Considers internal wall deposits	[35]
		Button					Not discussed	
		CIP-10l						
		GSP						
		IOM sampler				Filter and cassette considered		
	Inhalable, respirable and thoracic	Respicon	Wood	64 inhalable dust pairs, 53 thoracic dust pairs, 66 thoracic dust pairs	Gravimetric	Filter weights only considered	Not discussed	[40]
		IOM plastic sampler						
		SKC Aluminium cyclone						
		GK 2.69 cyclone						

Abbreviations: MCE: Mixed Cellulose Ester, PTFE: Polytetrafluoroethylene, PVC: Polyvinyl chloride.

## References

[1] Orenstein A.J. (1960). Proceedings of Pneumoconiosis Conference, Johannesburg 1959.

[2] Soderholm S.C. (1989). Proposed international conventions for particle size-selective sampling. Ann. Occup. Hyg..

[3] ISO (1995). ISO 7708:1995 Air Quality: Particle Size Fraction Definitions for Health-Related Sampling.

[4] Bevan R., Ashdown L., McGough D., Huici-Montagud A., Levy L. (2017). Setting evidence-based occupational exposure limits for manganese. Neurotoxicology.

[5] OSHA (2018). Final Rule to Protect Workers from Beryllium Exposure. https://www.osha.gov/berylliumrule/.

[6] Committee for Risk (2018). Assessment Opinion on Scientific Evaluation of Occupational Exposure Limits for Nickel and Its Compounds. https://echa.europa.eu/documents/10162/13641/nickel_opinion_en.pdf/9e050da5-b45c-c8e5-9e5e-a1a2ce908335.

[7] Waters M., McKernan L., Maier A., Jayjock M., Schaeffer V., Brosseau L. (2015). Exposure Estimation and Interpretation of Occupational Risk: Enhanced Information for the Occupational Risk Manager. J. Occup. Environ. Hyg..

[8] Kuempel E.D., Geraci C.L., Schulte P.A. (2012). Risk Assessment and Risk Management of Nanomaterials in the Workplace: Translating Research to Practice. Ann. Occup. Hyg..

[9] Verpaele S., Brisson M. (2019). Impact of the Detection and Quantitation Limits on the Analytical Feasibility of Measuring the European Chemicals Agency Risk Assessment Committee’s Recommendations for Occupational Exposure Limit Values for Nickel and Its Compounds in the Workplace. Detection Limits in Air Quality and Environmental Measurements.

[10] Verpaele S., Butler O. Measurements of Trace Metals and Metalloids. Deliberations from an ASTM Workshop. https://synergist.aiha.org/202008-trace-metals-metalloids.

[11] Vaughan N.P., Chalmers C.P., Botham R.A. (1990). Field comparison of personal samplers for inhalable dust. Ann. Occup. Hyg..

[12] Vincent J.H., Tsai P.-J., Warner J.S. (1995). Sampling of inhalable aerosol with special reference to speciation. Analyst.

[13] Vinzents P.S., Thomassen Y., Hetland S. (1995). A method for establishing tentative occupational exposure limits for inhalable dust. Ann. Occup. Hyg..

[14] Aitken R., Donaldson R. (1996). Large Particle and in Wall Deposition Effects Inhalable Samplers.

[15] Tsai P.J., Vincent J.H., Wahl G.A., Maldonado G. (1996). Worker exposures to inhalable and total aerosol during nickel alloy production. Ann. Occup. Hyg..

[16] Wilsey P.W., Vincent J.H., Bishop M.J., Brosseau L.M., Greaves I.A. (1996). Exposures to Inhalable and “Total” Oil Mist Aerosol by Metal Machining Shop Workers. Am. Ind. Hyg. Assoc. J..

[17] Kenny L.C., Aitken R., Chalmers C., Fabriès J.F., Gonzalez-Fernandez E., Kromhout H., Lidén G., Mark D., Riediger G., Prodi V. (1997). A collaborative European study of personal inhalable aerosol sampler performance. Ann. Occup. Hyg..

[18] Kenny L.C., Bowry A., Crook B., Stancliffe J.D. (1999). Field testing of a personal size-selective bioaerosol sampler. Ann. Occup. Hyg..

[19] Ogden T., Birkett J., Walton W. (1997). The Human Head as a Dust Sampler. Inhaled Particles IV.

[20] Demange M., Görner P., Elcabache J.-M., Wrobel R. (2002). Field comparison of 37-mm closed-face cassettes and IOM samplers. Appl. Occup. Environ. Hyg..

[21] Lidén G., Melin B., Lidblom A., Lindberg K., Norén J.O. (2000). Personal sampling in parallel with open-face filter cassettes and IOM samplers for inhalable dust-implications for occupational exposure limits. Appl. Occup. Environ. Hyg..

[22] Görner P., Wrobel R., Micka V., Skoda V., Denis J., Fabriès J.F. (2001). Study of fifteen respirable aerosol samplers used in occupational hygiene. Ann. Occup. Hyg..

[23] Teikari M., Linnainmaa M., Laitinen J., Kalliokoski P., Vincent J., Tiitta P., Raunemaa T. (2003). Laboratory and field testing of particle size-selective sampling methods for mineral dusts. AIHA J. Sci. Occup. Environ. Health Saf..

[24] Aizenberg V., Choe K., Grinshpun S.A., Willeke K., Baron P.A. (2001). Evaluation of personal aerosol samplers challenged with large particles. J. Aerosol Sci..

[25] BSI (1993). BS EN 481:1993 Workplace Atmospheres: Size Fraction Definitions for Measurement of Airborne Particles.

[26] EN 482 (2012). 2012 Workplace Exposure—General Requirements for the Performance of Procedures for the Measurement of Chemical Agents.

[27] EN 13205-5 (2014). Workplace Exposure—Assessment of Sampler Performance for Measurement of Airborne Particle Concentrations—Part 5: Aerosol Sampler Performance Test and Sampler Comparison Carried out at Workplaces.

[28] Linnainmaa M., Laitinen J., Leskinen A., Sippula O., Kalliokoski P. (2008). Laboratory and field testing of sampling methods for inhalable and respirable dust. J. Occup. Environ. Hyg..

[29] Hawley Blackley B., Gibbs J.L., Cummings K.J., Stefaniak A.B., Park J.Y., Stanton M., Virji M.A. (2019). A field evaluation of a single sampler for respirable and inhalable indium and dust measurements at an indium-tin oxide manufacturing facility. J. Occup. Environ. Hyg..

[30] Anthony T.R., Cai C., Mehaffy J., Sleeth D., Volckens J. (2017). Performance of prototype high-flow inhalable dust sampler in a livestock production facility. J. Occup. Environ. Hyg..

[31] Chisholm W.P., Lee T., Slaven J.E., Nelson J., Harper M. (2012). Comparison of Filter and Wall Deposits from Samplers Used to Collect Airborne Lead-Containing Dusts at Field Sites. Aerosol Sci. Technol..

[32] Harper M., Akbar M.Z., Andrew M.E. (2004). Comparison of wood-dust aerosol size-distributions collected by air samplers. J. Environ. Monit..

[33] Kauffer E., Wrobel R., Görner P., Rott C., Grzebyk M., Simon X., Witschger O. (2010). Site Comparison of Selected Aerosol Samplers in the Wood Industry. Ann. Occup. Hyg..

[34] Campopiano A., Basili F., Angelosanto F., Cannizzaro A., Olori A., Ramires D., Iannò A., Angelici L. (2016). Field comparison of two inhalable samplers used in Italy to measure the wood dust exposure. Int. J. Occup. Environ. Health.

[35] Lee T., Harper M., Slaven J.E., Lee K., Rando R.J., Maples E.H. (2011). Wood dust sampling: Field evaluation of personal samplers when large particles are present. Ann. Occup. Hyg..

[36] Kock H., Civic T., Koch W. (2015). Beryllium Concentrations at European Workplaces: Comparison of “Total” and Inhalable Particulate Measurements. Ann. Occup. Hyg..

[37] De Vocht F., Hirst A., Gardner A. (2009). Application of PUF Foam Inserts for Respirable Dust Measurements in the Brick-Manufacturing Industry. Ann. Occup. Hyg..

[38] Notø H.P., Nordby K.-C., Eduard W. (2016). Relationships between Personal Measurements of “Total” Dust, Respirable, Thoracic, and Inhalable Aerosol Fractions in the Cement Production Industry. Ann. Occup. Hyg..

[39] Dufresne A., Dion C., Viau S., Cloutier Y., Perrault G. (2009). Beryllium aerosol characteristics in the magnesium and aluminum transformation industry in Quebec: A comparison of four different sampling methodologies. J. Occup. Environ. Hyg..

[40] Rando R., Poovey H., Mokadam D., Brisolara J., Glindmeyer H. (2005). Field performance of the RespiCon for size-selective sampling of industrial wood processing dust. J. Occup. Environ. Hyg..

[41] Tsai C.-J., Chang C.-S., Chen S.-C., Chen P., Shih T.-S., Pui D.Y.H., Karasev V.V., Onischuk A.A., Li S.-N. (2008). Laboratory and Field Tests of a Novel Three-Stage Personal Dust Sampler for Sampling Three Dust Fractions Simultaneously. Aerosol Sci. Technol..

[42] Skaugset N.P., Ellingsen D.G., Notø H., Jordbekken L., Thomassen Y. (2013). Intersampler Field Comparison of Respicon^®^, IOM, and Closed-Face 25-mm Personal Aerosol Samplers During Primary Production of Aluminium. Ann. Occup. Hyg..

[43] Reynolds S.J., Nakatsu J., Tillery M., Keefe T., Mehaffy J., Thorne P.S., Donham K., Nonnenmann M., Golla V., O’shaughnessy P. (2009). Field and Wind Tunnel Comparison of Four Aerosol Samplers Using Agricultural Dusts. Ann. Occup. Hyg..

[44] Kriech A.J., Osborn L.V., Wissel H.L., Kurek J.T., Sweeney B.J., Peregrine C.J.G. (2004). Total versus inhalable sampler comparison study for the determination of asphalt fume exposures within the road paving industry. J. Environ. Monit..

[45] Lee E.G., Grimson P.J., Chisholm W.P., Kashon M.L., He X., L’Orange C., Volckens J. (2019). Performance evaluation of disposable inhalable aerosol sampler at a copper electrorefinery. J. Occup. Environ. Hyg..

[46] de Vocht F., Huizer D., Prause M., Jakobsson K., Peplonska B., Straif K., Kromhout H. (2006). Field comparison of inhalable aerosol samplers applied in the european rubber manufacturing industry. Int. Arch. Occup. Environ. Health.

[47] Deygout F., Le Coutaller P. (2010). Field sampling investigations within the road paving industry. J. Occup. Environ. Hyg..

[48] Jeong J.Y., Park J.S., Kim P.G. (2016). Characterization of Total and Size-Fractionated Manganese Exposure by Work Area in a Shipbuilding Yard. Saf. Health Work.

[49] Lee E.G., Chisholm W.P., Burns D.A., Nelson J.H., Kashon M.L., Harper M. (2014). Comparison of lead and tin concentrations in air at a solder manufacturer from the closed-face 37-mm cassette with and without a custom cellulose-acetate cassette insert. J. Occup. Environ. Hyg..

[50] L’Orange C., Anderson K., Sleeth D., Anthony T.R., Volckens J. (2016). A Simple and Disposable Sampler for Inhalable Aerosol. Ann. Occup. Hyg..

[51] Borsh F.B., Sleeth D.K., Handy R.G., Pahler L.F., Andrews R., Ashley K. (2019). Evaluation of a 25-mm disposable sampler relative to the inhalable aerosol convention. J. Occup. Environ. Hyg..

[52] Committee for Risk Assessment (2018). Annex XV Restriction Report for Five Cobalt Salts.

[53] AGS and BAuA (2018). Ausschuss für Gefahrstoffe—AGS, BAuA Begründung zu Antimontrioxid und Antimontrisulfid (A-Staub) in TRGS 900. https://www.baua.de/DE/Angebote/Rechtstexte-und-Technische-Regeln/Regelwerk/TRGS/pdf/900/900-antimontrioxid-antimontrisulfid.pdf?__blob=publicationFile&v=2.

[54] European Committee for Standardization (2005). Workplace Atmospheres—Guidance for Sampling of Inhalable, Thoracic and Respirable Aerosol Fractions, CEN TR 15230.

